# Risk of Subsequent Hip Fractures across Varying Treatment Patterns for Index Vertebral Compression Fractures

**DOI:** 10.3390/jcm13164781

**Published:** 2024-08-14

**Authors:** Andy Ton, Jennifer A. Bell, William J. Karakash, Thomas D. Alter, Mary Kate Erdman, Hyunwoo Paco Kang, Emily S. Mills, Jonathan Mina Ragheb, Mirbahador Athari, Jeffrey C. Wang, Ram K. Alluri, Raymond J. Hah

**Affiliations:** 1 Department of Orthopaedic Surgery, Keck School of Medicine, The University of Southern California, Los Angeles, CA 90033, USA; andy.t.ton@gmail.com (A.T.); wkarakas@usc.edu (W.J.K.);; 2 Department of Orthopaedic Surgery, Mayo Clinic, Rochester, MN 55905, USA; 3 Department of Orthopaedic Surgery, University of Chicago Medical Center, Chicago, IL 60612, USA; 4 Department of Orthopaedic Surgery, Kaiser Permanente Bernard J. Tyson School of Medicine, Los Angeles, CA 91101, USA

**Keywords:** vertebral compression fracture, fragility fracture, osteoporosis, hip fracture, anti-osteoporotic treatment, bisphosphonates

## Abstract

**Introduction:** Vertebral compression fractures (VCFs) pose a considerable healthcare burden and are linked to elevated morbidity and mortality. Despite available anti-osteoporotic treatments (AOTs), guideline adherence is lacking. This study aims to evaluate subsequent hip fracture incidence after index VCF and to elucidate AOT prescribing patterns in VCF patients, further assessing the impact of surgical interventions on these patterns. **Materials and Methods:** Patients with index VCFs between 2010 and 2021 were identified using the PearlDiver database. Diagnostic and procedural data were recorded using International Classification of Diseases (ICD-9, ICD-10) and Current Procedural Terminology (CPT) codes. Patients under age 50 and follow-up <one year following index VCF were excluded. Patients were categorized based on whether they received AOT within one year, preceding and after index VCF, and were subsequently propensity-matched 1:3 based on age, sex, and Elixhauser Comorbidity Index (ECI) score to compare hip fracture incidence following index VCF. Sub-analysis was performed for operatively managed VCFs (kyphoplasty/vertebroplasty). Statistical tests included Chi-squared for categorical outcomes, and Kruskal–Wallis for continuous measures. **Results:** Of 637,701 patients, 72.6% were female. The overall subsequent hip fracture incidence was 2.6% at one year and 12.9% for all-time follow-up. Propensity-matched analysis indicated higher subsequent hip fracture rates in patients initiated on AOT post-index VCF (one year: 3.8% vs. 3.5%, *p* = 0.0013; all-time: 14.3% vs. 13.0%, *p* < 0.0001). **Conclusions:** The study reveals an unexpected increase in subsequent hip fractures among patients initiated on AOT post-index VCF, likely due to selection bias. These findings highlight the need for refined osteoporosis-management strategies to improve guideline adherence, thereby mitigating patient morbidity and mortality.

## 1. Introduction

Fragility fractures represent a significant source of preventable disability and mortality worldwide [1,2]. In patients with osteoporosis, structural bone deficits significantly lower the threshold to mechanical failure. Functional impairments associated with advanced age, as seen in osteoporosis, further exponentiates the risk of fragility fractures within this patient demographic [3]. In consideration of an aging population, both prevalence and incidence of osteoporotic fractures are only expected to increase [4]. This trend poses a growing challenge to healthcare systems globally, necessitating a re-evaluation of current prevention and treatment strategies.

For initial occurrences, osteoporotic vertebral compression fractures (VCFs) are the most prevalent, with hip fractures being predominant drivers of fragility fracture recurrence [5,6,7]. This pattern highlights the importance of early intervention following index VCF to mitigate the potential risk of incurring subsequent fractures with significant morbidity and mortality. While anti-osteoporotic treatment (AOT) is appropriately indicated after index fragility fractures to prevent recurrence, extensive studies have highlighted poor provider adherence to treatment guidelines [1,2,3,8]. This discrepancy between evidence-based recommendations and real-world practice patterns represents a significant opportunity for improving patient care and outcomes.

The inconsistencies in osteoporosis treatment and the preventable nature of fragility fractures are well-recognized issues. Although a substantial body of literature exists to highlight AOT efficacy for fragility fracture prevention—within the United States (US)—variations in provider adherence to treatment guidelines following index VCF and their presumptive impact on fracture recurrence are inadequately described [9,10,11]. This knowledge gap hinders the development of optimized treatment strategies and potentially leaves patients at unnecessary risk. Accounting for the high morbidity and mortality risk conferred by fragility-based hip fractures and the leading incidence of VCFs amongst osteoporotic fractures overall, the objective of this study is to determine the incidence and outline the risk of subsequent hip fractures following index VCF as a function of AOT adherence [12,13,14]. Further investigation of these relationships could identify directions for improvement and refine prevention strategies for a leading cause of disability and mortality. Ultimately, this research seeks to contribute to the broader goal of reducing the burden of fragility fractures on individuals and healthcare systems alike.

## 2. Materials and Methods

### 2.1. Study Design and Data Source

The PearlDiver Mariner Database (PearlDiver Technologies Inc., Colorado Springs, CO, USA) was queried for all patients who had a pathological vertebral fracture from January 2010 to October 2021. All relevant diagnoses and procedures were identified using International Classification of Diseases (ICD-9 and ICD-10) and Current Procedural Terminology (CPT) codes. Anti-osteoporotic treatment (AOT) was identified using National Drug Codes (NDC) for anti-resorptive (bisphosphonates, denosumab, calcitonin) and anabolic medications (teriparatide). 

### 2.2. Study Participants

**Figure 1:**
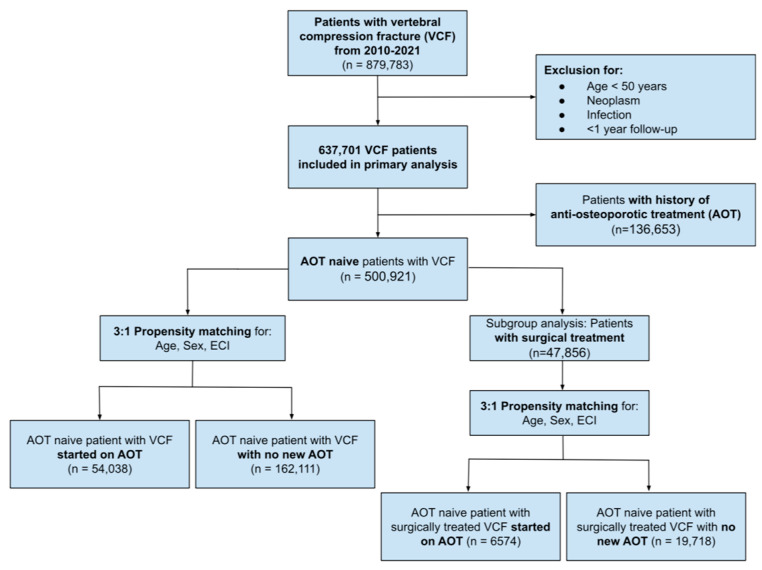
Patient-selection flowchart. VCF = vertebral compression fracture, AOT = anti-osteoporotic treatment, ECI = Elixhauser Comorbidity Index.

Patient demographics including age, gender, and comorbidities were recorded, wherein the the Elixhauser Comorbidity Index (ECI) was used to quantify degree of patient comorbidities. Patients were excluded if they were (1) under 50 years of age (2) had less than one-year follow-up from index VCF and/or (3) had a concurrent diagnosis of neoplasm or infection. Based on expert consensus, age >50 years was selected as an inclusion criteria for this study given its role as an established predictor of substantially increased fragility fracture risk amongst patients with osteoporosis [15,16,17]. A patient-selection flowchart diagram is depicted in Figure 1. Institutional Review Board (IRB) approval was not required for this study per the National Institutes of Health (NIH) research guidelines, as the study utilizes a national database that has been de-identified and furthermore does not involve any form of data collection gathered through interaction/intervention with human subjects [18]. 

### 2.3. Treatment Groups

Patients were grouped in one of two treatment categories based on AOT treatment within one year—both prior to and after index VCF. Prior to index VCF, patients were divided into one of the following treatment groups: (Group 1) prior AOT—patients treated with AOT; (Group 2) treatment naive—patients who did not receive AOT. After index VCF, treatment naive patients were further separated into one of two treatment groups: (Group 3) newly treated—treatment naive patients initiated on AOT following index VCF; (Group 4) untreated—treatment naive patients who were not initiated on AOT following index VCF. Patients were furth subcategorized on whether or not treatment included surgical intervention (ie vertebroplasty or kyphoplasty). 

In order to estimate the effect of a treatment by accounting for the covariates that predict receiving the treatment, a 1:3 propensity-matched analysis adjusting for age, sex, and ECI identified two groups eligible for subsequent analysis; treatment naive patients initiated on AOT following index VCF (Group 3) and treatment naive patients without AOT following index VCF (Group 4). A sub-group propensity-matched analysis was also completed for groups 3 and 4 who received surgical treatment following VCF. Propensity matching was employed in an effort to reduce bias due to confounding variables that could be found in an estimate of the treatment effect obtained from simply comparing outcomes among units that received the treatment versus those that did not. Primary outcome measures included one-year and all-time subsequent hip fracture incidence following index VCF.

### 2.4. Statistical Analysis

The Shapiro–Wilks test was used to assess data normality, followed by non-parametric analyses given the non-normal distribution of the data. Kruskal–Wallis testing was used to compare age distributions and ECI scores across groups, while chi-squared analysis was conducted to assess differences in categorical variables such as sex and subsequent hip fracture incidence. Additional analysis was conducted to identify relationships between age, gender, and comorbidity burden with subsequent hip fracture risk. All statistical analyses were performed using R statistical software integrated within PearlDiver Bellwether software. 

## 3. Results

A total of 637,701 patients with VCF were included in the analysis. The majority of patients were female (72.6%) with age 70–74 being the most common age group (37.6%). Of these patients, 68,535 (10.7%) were treated operatively (Table S1). 

Overall, subsequent hip fracture incidence for all VCF patients was 2.6% (16,795 patients) at one-year follow-up and 12.9% (82,201 patients) all-time. The majority of patients (555,500; 87.1%) did not experience a subsequent hip fracture during the entire follow-up period. When stratified by prior AOT status, patients with prior AOT had a slightly higher all-time hip fracture rate (13.9%) compared to those without prior AOT (12.6%). Patients who were initiated on new AOT after their VCF had slightly higher rates of subsequent hip fracture both at one year (3.8% vs. 3.4%) and all-time (14.3% vs. 12.4%) compared to those who did not receive new AOT (Table S2). 

Amongst surgically treated patients, one-year and all-time subsequent hip fracture incidence was 2.8% and 10.9%, respectively. Within the surgically treated group, those with prior AOT had a higher all-time hip fracture rate (11.2%) compared to those without prior AOT (10.8%) (Table S2). Age was significantly associated with hip fracture risk (*p* < 0.0001). The percentage of hip fracture patients increased with age, from 1.8% in the 50–54 age group to 48.0% in the 70–74 age group and 30.1% in the 75–79 group. Females represented 79.8% of those who experienced a subsequent hip fracture, compared to 71.5% of those who did not (*p* < 0.0001). A similar difference was also observed in the surgically treated group (77.8% vs. 76.0%, *p* < 0.0001). ECI was significantly higher in patients who experienced a subsequent hip fracture. In the overall VCF population, the mean ECI score for patients with a hip fracture was 8.1 ± 4.2, vs. 4.6 ± 3.6 for those without (*p* < 0.0001). The difference was similar but with higher magnitude in the surgically treated group (9.2 ± 4.3 vs. 6.9 ± 4.1, *p* < 0.0001). An overview of patient characteristics in relation to subsequent hip fractures rates is outlined in Table S3.

Propensity-matched analysis adjusted for age, gender, and ECI identified 1:3 matched cohorts of 54,038 patients initiated on new AOT following VCF and 162,111 without AOT treatment (Table S4). Patients initiated on AOT were found to have higher rates of subsequent hip fracture at one year (3.8% vs. 3.5%; *p* < 0.002) and all-time (14.3% vs. 13.0%; *p* < 0.0001). Within the subgroup analysis of patients with operatively managed VCFs, there was no difference in hip fracture rates at both timepoints in treatment-naive patients initiated on AOT and those who were deferred AOT. The all-time hip fracture rate was 11.2% for those initiated on new AOT compared to 10.8% for those not initiated on new AOT (*p* = 0.328). Similarly, the one-year hip fracture rates were 2.7% and 2.5%, respectively (*p* = 0.420) (Table S5).

## 4. Discussion

Amongst 637,701 patients identified with a primary diagnosis of VCF, 72.6% were female and the most common age group was 70–74 years. These demographic characteristics parallel population-based studies across the United States and Europe, wherein osteoporotic VCF incidence is two-fold greater in women and increasing age is associated with significantly greater risk of VCFs [19,20]. This gender disparity and age distribution highlight the importance of targeted screening and prevention strategies for these high-risk groups. The peak incidence in the 70–74 age group suggests a critical window for intervention that healthcare providers should be aware of when managing older patients.

Conversely, only 10.1% of our study cohort underwent surgical management for their VCF. Within the surgically treated cohort, subsequent hip fracture incidence was 2.8% at one year and 10.9% for all-time, similar to rates seen across the overall VCF cohort with 2.6% and 12.9% for one year and all-time, respectively. Although marginal, the discrepancy in all-time subsequent hip fracture incidence between the surgical and overall cohort is likely premised on the severity of the pathology. Most cases of VCF are initially treated and resolved with conservative management, while operative strategies are commonly reserved for patients who fail conservative therapy or sustain highly deforming VCFs, both which may result from higher severity of osteoporosis [21,22,23]. The higher subsequent fracture rates in the surgical group likely reflect this selection bias towards more severe cases. Differences in all-time hip fracture rates in our study are therefore likely owed to greater disease burden in patients requiring surgical intervention, rather than a direct effect of the surgical intervention itself [24].

Analysis of demographic characteristics found female sex, increasing age, and ECI scores to be significant predictors of subsequent hip fractures following index VCFs. These findings align with established risk factors for osteoporotic fractures and highlight the multifactorial nature of fracture risk. Ensrud (2013) conducted a well-cited review showing women over age 65 across the United States accounted for 74% of fragility fractures and 89% of associated care-related costs [25]. This disproportionate impact on older women underscores the need for targeted prevention strategies in this high-risk group. The growing impact of osteoporotic VCFs within these aging populations, especially women, can be explained by an associated increase in their comorbidity burden—a natural function of increasing age. The strong association between ECI scores and fracture risk emphasizes the importance of comprehensive assessment and management in fracture prevention. These findings suggest that a holistic approach to patient care, considering multiple risk factors beyond bone density alone, may be crucial in effectively reducing fracture risk in vulnerable populations.

Notably, our study found a paradoxically higher rate of subsequent hip fractures following index VCF in patients newly initiated on AOT compared to those who were not. Differences in subsequent hip fracture rates further increased when comparing one-year and all-time incidence. It is unlikely that these findings stem from the treatment effect of AOTs, but as a reflection of inconsistent AOT prescribing practices, wherein AOT is selectively prescribed for pathologies perceived to be of higher severity. Though seemingly intuitive, the propensity to initiate AOT only on patients with seemingly higher risk of subsequent fractures may be inadvertently leading to undertreatment of patients with lower—but nonetheless—treatable risk. For high-risk patients, a more intensive approach combining pharmacological treatment with lifestyle modifications, fall prevention strategies, and close follow-up may be necessary to effectively reduce fracture risk. Clinicians across the U.S. should also consider initiating AOT in all patients with fragility fractures, regardless of perceived severity, to mitigate risk of subsequent fractures in a broader patient population. For example, the implementation of a fracture liaison service (FLS) for treatment of patients with fragility fractures across Australia, Netherlands, and England saw significant reductions in subsequent fragility fracture incidence and mortality [26,27,28]. The FLS was designed with the intention to standardize fragility fracture management, such that treatment was no longer based on provider discretion but rather based on objective parameters used to guide appropriate treatment protocols. As such, the results in our study may appear anomalous but are likely confounded by nonuniform adherence to osteoporosis-management guidelines amongst providers throughout the US; the constellation of these findings allude to both the continued need for refined treatment protocols and to the prospective benefit from implementing a standardized approach for managing patients with fragility fractures.

Delving further, the lack of adherence to clinical guidelines for osteoporosis management has been well-documented and shown by declining AOT prescriptions despite increasing incidence [29]. Many asymptomatic patients are diagnosed incidentally or present with adequately controlled pain—these factors often misguide clinicians into deferring AOT in these patients [30]. Bartalena et al. performed a systematic review of 12 radiographic studies to evaluate reporting rates for incidentally diagnosed VCFs. Across these studies, radiologists had a mean reporting rate of 27.4% with more recently conducted studies demonstrating significantly lower rates [31]. Freedman et al. performed a retrospective review of 156 patients showing that only 39% patients were referred for a dual-energy X-ray absorptiometry (DEXA) scan, 35% to an endocrinologist, and only 38% were given active treatment [32]. Taken as a constellation, these findings shed light on a multilayered issue underlying inadequate osteoporosis management at a systematic level.

The role of patient education, activation, and adherence in osteoporosis management cannot be overstated. The asymptomatic nature of osteoporosis, like other asymptomatic chronic conditions, often leads to poor treatment adherence until a fracture occurs. This highlights the need for improved patient education programs and regular follow-ups to ensure treatment compliance. This prompts further development of newer, more convenient dosing regimens for AOT, which may help improve long-term adherence and, consequently, fracture prevention outcomes.

Further subanalysis of AOT groups within operatively treated patients, however, removed discrepancies in subsequent hip fracture incidence at both one year and all-time. Premised on the notion that AOT was selectively prescribed in patients with more severe VCFs, this finding implies a significant benefit with operative intervention for increasingly severe pathologies. By reducing pain and reinstating early mobilization, percutaneous vertebral augmentation substantially reduces morbidity and mortality, especially in osteoporotic hip fractures wherein weight-bearing is imperative for prevention [33,34].

### 4.1. Limitations

Our study has several limitations by nature of its retrospective, observational design. One such example includes the effect of unaccounted confounders within our analysis. The presence of these variables likely influenced our findings to some degree, and represents a bias inherent to all observational studies that should be taken into consideration when interpreting the results. Additionally, while the use of ICD diagnostic codes is intended to standardize data reporting across institutions, findings herein were obtained using a secondary data source and thus remain subject to any variability in data-collection practices between institutions. 

Moreover, in using a national data registry, our outcome measures were largely confined to binarized data elements which lack the granularity to characterize individualized disease features and severity. Furthermore, although we identified an underlying explanation for paradoxically increased hip fractures observed with AOT-initiated patients, we also acknowledge that this finding is likely a reflection of underlying selection bias represented by higher grade VCFs and osteoporosis severity within this cohort. While unable to control for diagnosis severity, we sought to adjust for other confounding group differences by using ECI scores to quantify comorbidity burden and incorporating them into a propensity-matched analysis.

### 4.2. Future Directions

Prospective studies using quantitative diagnostics like DEXA scans are needed to clarify the relationship between osteoporosis severity, AOT initiation, and subsequent fracture risk, potentially explaining our observed paradoxical results and informing optimal AOT timing. Concurrently, developing refined risk stratification tools could enable more personalized treatment approaches; while investigating new strategies such as combination or sequential therapies may improve fracture prevention, especially in high-risk patients. Research must also address the gap between treatment guidelines and clinical practice, evaluating methods to improve healthcare providers’ adherence to guidelines and assessing the impact of patient education on treatment adherence and fracture prevention. These efforts collectively aim to develop a comprehensive approach to osteoporosis management, potentially improving patient outcomes and reducing the burden of fragility fractures.

## 5. Conclusions

Our analysis reveals higher rates of hip fractures in patients with surgically treated VCF and those initiated on AOT following VCF, likely reflecting clinician selection bias towards treating patients with more severe osteoporosis. This paradoxical relationship underscores the need for standardized osteoporosis-management protocols following fragility fractures. Addressing the persistent gap between treatment guidelines and clinical practice through improved provider education and patient engagement strategies is essential. Further research using quantitative diagnostics like DEXA scans is crucial to elucidate the relationship between osteoporosis severity, AOT initiation timing, and fracture outcomes. Outlining these relationships will identify potential areas where management strategies for osteoporosis and fragility fractures can be improved. By addressing these critical needs within the field, we can work towards a more comprehensive and effective strategy for osteoporosis management, potentially improving patient outcomes, reducing healthcare costs, and enhancing the quality of life for patients with osteoporosis. 

## Data Availability

The data presented in this study are available on request from the corresponding author.

## Supplementary Materials

The following supporting information can be downloaded at: https://www.mdpi.com/article/10.3390/jcm13164781/s1, Table S1: Demographics of Patients with Vertebral Compression Fracture; Table S2: Hip Fracture Rates Following VCF; Table S3: Patient Characteristics Based on Presence of Succeeding Hip Fracture Following VCF; Table S4: Demographics of AOT Naïve Patients with VCF Before and After Matching; Table S5: Hip Fracture Rates Following VCF in Matched Cohorts of AOT Naive Patients.
