# Supplementary material for: Risk of Subsequent Hip Fractures across Varying Treatment Patterns for Index Vertebral Compression Fractures
Source: J Clin Med. 2024 Aug 14;13(16):4781. doi: 10.3390/jcm13164781 (PMC11355522; doi:10.3390/jcm13164781)
Supplement: Supplementary file 1 [file jcm-13-04781-s001.zip › jcm-3107255-supplementary.pdf]

Table S1. Demographics of Patients with Vertebral Compression Fracture

	All Patients	Surgical Treatment	History of AOT	AOT Naïve	AOT Naïve		p-value
					Initiated AOT	No New AOT	
Total	637701	68535	136653	500921	54049	446872	
Age (mean ± SD)	70.5 ± 6.4	72.9 ± 7.4	72.3 ± 6.4	70.0 ± 7.3	70.2 ± 6.8	69.9 ± 7.4	<0.001
ECI (mean ± SD)	4.6 ± 3.6	6.06 ± 4.0	5.1± 3.7	4.4 ± 3.6	4.2 ± 3.5	4.4 ± 3.6	<0.001
Female (% of total)	72.6%	78.4%	89.7%	67.9%	78.4%	66.6%	<0.001

Abbreviations: VCF, Vertebral Compression Fractures; AOT, Anti-Osteoporotic Treatment

Table S2. Hip Fracture Rates Following VCF

	**Hip Fracture (1 Year)**	**Hip Fracture (All-Time)**	**No Fracture (All-Time)**
VCF	16795 (2.6%)	82201 (12.9%)	555500 (87.1%)
Prior AOT	5258 (3.8%)	19019 (13.9%)	117634 (86.1%)
No Prior AOT	17347 (3.5%)	63162 (12.6%)	437759 (87.4%)
No New AOT	15303 (3.4%)	55422 (12.4%)	391450 (87.6%)
Initiated New AOT Started	2044 (3.8%)	7740 (14.3%)	46309 (85.7%)
VCF with Surgical Treatment	1942 (2.8%)	7493 (10.9%)	61042 (89.1%)
Prior AOT	659 (3.2%)	2320 (11.2%)	18341 (88.8%)
No Prior AOT	1283 (2.7%)	5170 (10.8%)	42686 (89.2%)
No New AOT	1609 (3.9%)	4469 (10.8%)	36790 (89.2%)
Initiated New AOT Started	180 (2.7%)	741 (11.2%)	5856 (88.8%)

Abbreviations: VCF, Vertebral Compression Fractures; AOT, Anti-Osteoporotic Treatment

Table S3. Patient Characteristics Based on Presence of Succeeding Hip Fracture Following VCF

	All VCF			VCF with Surgical Treatment		
	**Hip Fracture**	**No Hip Fracture**	***p* value**	**Hip Fracture**	**No Hip Fracture**	***p* value**
Age			<0.0001			<0.0001
50 to 54	1.8%	4.8%		1.1%	2.5%	
55 to 59	3.5%	7.2%		3.4%	5.2%	
60 to 64	5.2%	9.2%		4.9%	8.2%	
65 to 69	8.3%	11.7%		9.0%	12.0%	
70 to 74	48.0%	36.0%		24.4%	19.4%	
75 to 79	30.1%	26.2%		44.3%	36.2%	
80 and over	3.2%	4.8%		12.8%	16.4%	
Gender			<0.0001			<0.0001
Female	79.8%	71.5%		77.8%	76.0%	
Male	20.2%	28.5%		22.2%	24.0%	
ECI	8.1 ± 4.2	4.6 ± 3.6	<0.0001	9.2 ± 4.3	6.9 ± 4.1	< 0.0001

Abbreviations: VCF, Vertebral Compression Fractures

Table S4. Demographics of AOT Naïve Patients with VCF Before and After Matching

All VCF

	Unmatched Cohorts			Matched Cohorts		
	No New AOT	Initiated New AOT	p-value	No New AOT	Initiated New AOT	p-value
Total	446872	54049		162111	54038	
Age	69.93 ± 7.4	70.20 ± 6.8	<0.001	70.20 ± 6.8	70.20 ± 6.8	1
Gender			<0.001			1
Female	297741 (66.6%)	42373 (78.4%)		127095 (78.4%)	42365 (78.4%)	
Male	149131 (33.4%)	11676 (21.6%)		35016 (21.6%)	11673 (21.6%)	
ECI	4.5 ± 3.6	4.2 ± 3.5	<0.001	4.2 ± 3.5	4.2 ± 3.5	1

VCF with Surgical Treatment

	Unmatched Cohorts			Matched Cohorts		
	No New AOT	Initiated New AOT	p-value	No New AOT	Initiated New AOT	p-value
Total	41259	6597		19764	6589	
Age	72.56 ± 7.55	71.68 ± 7.6	<0.001	71.68 ± 7.6	71.68 ± 7.6	1
Gender			<0.001			1
Female	28670 (69.5%)	5060 (76.7%)		15168 (76.7%)	5057 (76.7%)	
Male	12589 (30.5%)	1537 (23.3%)		4596 (23.3%)	1532 (23.3%)	
ECI	6.0 ± 4.0	5.6 ± 3.8	<0.001	5.6 ± 3.8	5.6 ± 3.8	1

Abbreviations: VCF, Vertebral Compression Fractures; AOT, Anti-Osteoporotic Treatment

Table S5. Hip Fracture Rates Following VCF in Matched Cohorts of AOT Naive Patients

All VCF				
	**No New AOT**	**Initiated New AOT**	***p value***	**OR (95% CI)**
Fracture (1 Year)	5873 (3.5%)	2044 (3.8%)	0.0013	1.09 (1.03-1.15)
Fracture (All-Time)	21099 (13.0%)	7740 (14.3%)	<0.0001	1.12 (1.09-1.15)
No Fracture (All-Time)	141039 (87.0%)	46306 (85.7%)	<0.0001	0.89 (0.87-0.92)
**VCF with Surgical Treatment**				
	**No New AOT**	**Initiated New AOT**	***p value***	**OR (95% CI)**
Fracture (1 Year)	499 (2.5%)	177 (2.7%)	0.420	1.08 (0.91-1.28)
Fracture (All-Time)	2129 (10.8%)	739 (11.2%)	0.328	1.04 (0.96-1.13)
No Fracture (All-Time)	17635 (89.2%)	5850 (88.8%)	0.328	0.96 (0.87-1.04)

Abbreviations: VCF, Vertebral Compression Fractures; AOT, Anti-Osteoporotic Treatment
